# COVID-19 distributes socially in China: A Bayesian spatial analysis

**DOI:** 10.1371/journal.pone.0267001

**Published:** 2022-04-20

**Authors:** Di Peng, Jian Qian, Luyi Wei, Caiying Luo, Tao Zhang, Lijun Zhou, Yuanyuan Liu, Yue Ma, Fei Yin

**Affiliations:** 1 The First People’s Hospital of Shuangliu District, Chengdu, Sichuan, China; 2 West China School of Public Health and West China Fourth Hospital, Sichuan University, Chengdu, Sichuan, China; Institute for Advanced Sustainability Studies, GERMANY

## Abstract

**Purpose:**

The ongoing coronavirus disease 2019 (COVID-19) epidemic increasingly threatens the public health security worldwide. We aimed to identify high-risk areas of COVID-19 and understand how socioeconomic factors are associated with the spatial distribution of COVID-19 in China, which may help other countries control the epidemic.

**Methods:**

We analyzed the data of COVID-19 cases from 30 provinces in mainland China (outside of Hubei) from 16 January 2020 to 31 March 2020, considering the data of demographic, economic, health, and transportation factors. Global autocorrelation analysis and Bayesian spatial models were used to present the spatial pattern of COVID-19 and explore the relationship between COVID-19 risk and various factors.

**Results:**

Global Moran’s *I* statistics of COVID-19 incidences was 0.31 (*P*<0.05). The areas with a high risk of COVID-19 were mainly located in the provinces around Hubei and the provinces with a high level of economic development. The relative risk of two socioeconomic factors, the per capita consumption expenditure of households and the proportion of the migrating population from Hubei, were 1.887 [95% confidence interval (CI): 1.469~2.399] and 1.099 (95% CI: 1.053~1.148), respectively. The two factors explained up to 78.2% out of 99.7% of structured spatial variations.

**Conclusion:**

Our results suggested that COVID-19 risk was positively associated with the level of economic development and population movements. Blocking population movement and reducing local exposures are effective in preventing the local transmission of COVID-19.

## Introduction

Pneumonia caused by a novel coronavirus was identified in Wuhan, China, in December 2019 and then named coronavirus disease 2019 (COVID-19). Most patients experience mild to moderate respiratory symptoms, i.e., fever, fatigue, cough, and shortness of breath, but some patients with underlying health disorders, such as respiratory or cardiovascular diseases, can develop severe illness and even die [1]. A small proportion of COVID-19 cases are also reported without any symptoms but with positive viral nucleic acid test results [2]. COVID-19 transmits mainly via respiratory droplets and direct contact [3]. The incubation period of COVID-19 is within 14 days with a mean time of 5.2 days, and the mean basic reproductive number (R_0_) ranges from 1.4 to 3.9 [3–7]. Due to the long incubation period and high infectivity, COVID-19 cases can accumulate unidentified, and the high proportion of severe cases that come later may quickly overload the health system, consume local health resources, and cause large numbers of mortalities [8–11]. Therefore, the World Health Organization (WHO) declared COVID-19 a pandemic on 12 March 2020. As of 30 August 2020, the epidemic of COVID-19 had affected more than 200 countries and regions, with more than 20 million confirmed cases worldwide.

At present, several COVID-19 vaccines have been authorized for use throughout the world [12]. However, the lack of vaccine and coverage remains a challenge due to insufficient production and allocation. To date, public health intervention is still one of the most efficient ways to control the development of the COVID-19 pandemic [12, 13]. Studying the spatial patterns and influencing factors for COVID-19 is important to identify high-risk areas for further intervention and risk evaluation for local prevention and control strategy development. Additionally, health resources can be allocated in advance to prepare for the peak of incoming patients with severe illness. The distribution of COVID-19 shows significant spatial heterogeneity [14]. The occurrence and transmission of COVID-19 in the population may be influenced by natural, environmental, and social factors [14–18]. However, to date, there is a lack of comprehensive studies on quantifying the spatial pattern of COVID-19 and detecting the spatial impact of potential influencing factors on the COVID-19.

In our study, using spatial methods including spatial autocorrelation and Bayesian spatial models, we analyzed the data in 30 provinces in mainland China (outside of Hubei), and presented the spatial pattern of COVID-19. Our study detected the hot spots and cold spots of COVID-19, and explored the relationship between the risk of COVID-19 and socioeconomic factors. The purpose of this study was to determine high-risk areas of COVID-19 and explore potential influencing factors of COVID-19 to provide a basic understanding of COVID-19 for further public health interventions and resource allocations in other countries and areas.

## Methods

### Data sources

Data on the COVID-19 cases in 30 provinces in mainland China from 16 January 2020 to 31 March 2020, were collected from the National Health Commission of the People’s Republic of China. Hubei Province, where COVID-19 was first detected, was not included, as this study aimed to explore the socioeconomic and environmental risk factors after the import of the COVID-19 cases.

Four types of variables were collected for each province. Demographic variables included the total population, population density (PD), proportion of urban population at year-end (PUP), and percentage of illiterate population to total aged 15 and over (PIP). Economic variables included gross domestic product (GDP), foreign exchange earnings from international tourism (FEEFIT), and per capita consumption expenditure of households (PCCEOH). Health variables included the number of health care institutions (NOHCI), number of beds in health care institutions (NOB), medical technical personnel in health care institutions per 1000 persons (MTP), and beds of medical institutions per 1000 population (BOMI). Transportation variables included the passenger traffic (PT), passenger kilometers (PK), and proportion of the migrating population from Hubei from 16 January 2020 to 24 January 2020 (POMPFH). The POMPFH variable was provided by the Baidu Qianxi website [19]. The other variables were collected from the China Statistical Yearbook (2019).

### Data analysis

The cumulative incidences of COVID-19 in 30 provinces and descriptive statistics [range, mean, standard deviation (SD), median, and quartile] of variables were calculated. Depending on whether the variables were normally distributed, Pearson or Spearman correlation coefficients were used to estimate the crude correlation between the incidence of COVID-19 and provincial variables.

Global Moran’s *I* of the incidence of COVID-19 was calculated as follows to quantify spatial autocorrelation [20]:

$$I=\frac{n\cdot \sum_{i=1}^{n}\sum_{j=1}^{n}w_{ij}(y_{i}-\bar{y})(y_{j}-\bar{y})}{(\sum_{i=1}^{n}\sum_{j=1}^{n}w_{ij})\sum_{i=1}^{n}{\left(y_{i}-\bar{y}\right)}^{2}}$$ (1)

where *n* (*n* = 30) is the number of studied provinces; *y*_*i*_ and *y*_*j*_ are the incidence of COVID-19 in the *i*_th_ province and *j*_th_ province, respectively; $\bar{y}$ is the average incidence of COVID-19; ***w*** is the weight matrix to measure the adjacency relation of provinces, *w*_*ij*_ is 1 if provinces *i* and *j* are neighbors and 0 otherwise. Moran’s *I* ranges from -1 to 1, indicating the spatial autocorrelation of the incidence of COVID-19.

Bayesian spatial models were built to present the spatial pattern of COVID-19 and estimate the comprehensive relationship between the COVID-19 risk and variables. The Bayesian spatial model assumed that the reported counts of COVID-19 cases (*Y*_*i*_) for the *i*_th_ province followed a Poisson distribution with mean *λ*_*i*_ [21, 22]:

$$Y_{i}∼\mathrm{poisson}(\lambda_{i})$$ (2)

and

$$E(Y_{i})=\lambda_{i}=E_{i}\rho_{i}$$ (3)

where *E*(*Y*_*i*_) is the expected count of COVID-19 cases for each province, calculated by multiplying the overall incidence of COVID-19 of the 30 provinces by the population for each province during the study period; *ρ*_*i*_ is the relative risk (RR) of COVID-19 in the *i*_th_ province, and the log RR was modeled as

$$\log(\rho_{i})=b_{0}+\sum_{k=1}^{p}b_{k}x_{ki}+u_{i}+v_{i}$$ (4)

where *b*_0_ is the intercept, representing the average risk of COVID-19 in all the 30 provinces. *x*_*ki*_ is the *k*_*th*_ socioeconomic or environmental variable in the *i*_th_ province; *b*_*k*_ and $e^{b_{k}}$ are the regression coefficient and RR of the *k*_*th*_ variable, respectively; *u*_*i*_ is a spatially structured random effect, reflecting the spatial correlation between adjacent provinces; and *v*_*i*_ is a spatially unstructured random effect, which is caused by other nonspatial factors and can be interpreted as unknown or unobserved covariates at the provincial level. *u*_*i*_+*v*_*i*_ quantifies the total spatial effect and exp(*u*_*i*_+*v*_*i*_) is the spatial RR of the disease.

The integrated nested Laplace approximation (INLA) method was used to estimate the parameters of the Bayesian spatial model, and minimally informative priors were specified on the above random effects by default [23]. *b*_0_ and *b*_*k*_ were modeled using a vague prior followed the Gaussian distribution with mean zero and variance 10^6^. *u*_*i*_ was modeled using a conditional autoregressive specification (CAR) with mean zero and variance $\sigma_{u}^{2}$, and *v*_*i*_ was modeled as exchangeable with mean zero and variance $\sigma_{v}^{2}$. The precisions of *u*_*i*_ and *v*_*i*_ were specified as Gamma (1, 0.0005).

The spatial risk of COVID-19 for each province was identified based on the results of the Bayesian spatial model without covariables. According to the posterior distribution of *u*_*i*_+*v*_*i*_, 30 provinces were divided into hot, cold, and other spots. A province with the posterior probability $p(\exp(u_{i}+v_{i})>1|data)>0.9$ was defined as a hot spot; if its posterior probability $p(\exp(u_{i}+v_{i})>1|data)<0.1$, a province was defined as a cold spot; the remaining provinces were neither hot nor cold spots. In addition, the proportion of variance explained by *u*_*i*_ was calculated according to the posterior marginal distribution of $\sigma_{u}^{2}$ and $\sigma_{v}^{2}$ to quantify the relative importance of *u*_*i*_. The larger the proportion was, the more the variability was explained by *u*_*i*_. Then, according to the results of multivariate Bayesian spatial models, the relationship between COVID-19 and variables was explored, and the coefficients of variables were estimated.

Data were analyzed in R software (version 3.6.1, using “base”, “maptools”, “raster”, “spdep”, “rgdal”, “psych”, “ggplot2” and “INLA” packages).

## Results

### Descriptive analysis

**Fig 1:**
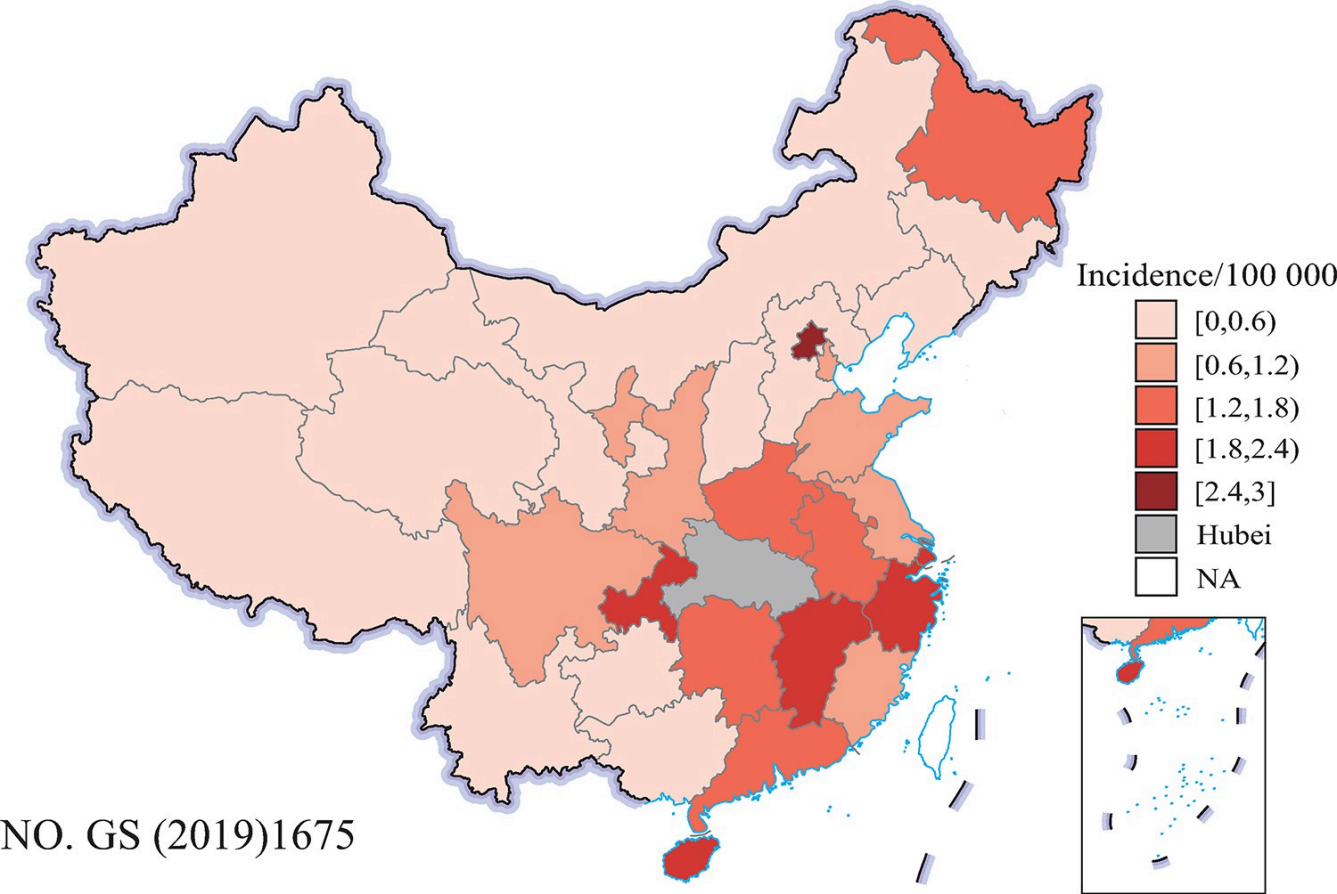
Cumulative incidence of COVID-19 (1/100000) in the 30 provinces in mainland China (outside of Hubei), from 16 January 2020 to 31 March 2020. The original map image was obtained from the China Ministry of Natural Resources(http://bzdt.ch.mnr.gov.cn/index.html).

From 16 January 2020 to 31 March 2020, a total of 13756 cases of COVID-19 were reported from the 30 provinces in mainland China (outside of Hubei). Fig 1 shows the spatial distribution of the cumulative incidence of COVID-19 at the provincial level. The incidence of COVID-19 ranged from 0.03/100000 (Tibet) to 2.70/100000 (Beijing). Provinces with high incidence were mainly located in eastern and southeastern China. The socioeconomic and environmental variables are presented in Table 1.

**Table 1:** Descriptive statistics of socioeconomic variables of the 30 provinces in mainland China (outsides of Hubei).

Variable	Mean (SD)	Min	*P*_25_	Median	*P*_75_	Max
PD (/square hectare)	4.66(7.18)	0.03	1.28	2.77	5.36	38.23
PUP (%)	59.98(11.98)	31.14	52.84	58.27	65.74	88.10
PIP (%)	5.98(6.13)	1.39	2.88	4.36	6.97	35.23
GDP (trillion yuan)	3.13(2.63)	0.17	1.37	2.42	3.94	10.77
FEEFIT (USD billion)	2.52(3.84)	0.03	0.66	1.39	3.05	20.51
PCCEOH (10000 yuan)	1.99(0.74)	1.15	1.59	1.71	2.10	4.34
NOHCI (10000 unit)	32.03(23.33)	4.45	18.92	27.74	36.42	85.09
NOB (10000 beds)	26.70(17.44)	1.68	15.99	24.75	33.11	60.85
MTP /1000 (person)	6.94(1.23)	5.27	6.29	6.75	7.34	11.88
BOMI /1000 (bed)	5.97(0.81)	4.37	5.42	6.04	6.55	7.21
PT (100 million persons)	5.45(3.78)	0.14	2.19	4.80	7.15	14.21
PKM (100 billion passenger-km)	0.74(0.53)	0.05	0.35	0.62	1.08	2.09
POMPFH (%)	3.32(4.16)	0.04	0.58	2.00	4.04	16.96

**Abbreviation**: PD, population, population density; PUP, proportion of urban population at year-end; PIP, the percentage of illiterate population to total aged 15 and over; GDP, gross domestic product; FEEFIT, foreign exchange earnings from international tourism; PCCEOH, per capita consumption expenditure of households, NOHCI: number of health care institutions, NOB: number of beds in health care institutions; MTP, medical technical personnel in health care institutions per 1000 persons; BOMI, beds of medical institutions per 1000 population; PT, passenger traffic; PKM, passenger-kilometers; POMPFH, proportion of the migrating population from Hubei from 16 January 2020 to 24 January 2020.

### Spearman correlation

Spearman correlation coefficients between the incidence of COVID-19 and variables ranged from -0.28 to 0.65 (S1 Table). Among the 13 variables, the incidence of COVID-19 was significantly associated with PD (*r* = 0.65, *P*<0.01), POMPFH (*r* = 0.57, *P*<0.01), PCCEOH (*r* = 0.54, *P*<0.01), PUP (*r* = 0.53, *P*<0.01), GDP (*r* = 0.46, *P*<0.05), and FEEFIT (*r* = 0.43, *P*<0.05). However, the correlations among the 6 socioeconomic variables were also significant. To reduce multicollinearity, one of the paired variables with a Spearman correlation coefficient greater than 0.7 was excluded. Thus, PUP and GDP were excluded in subsequent analyses.

### Spatial autocorrelation

The estimated Moran’s *I* statistic of the incidence of COVID-19 was 0.31 (*P*<0.05), indicating that a global spatial autocorrelation of COVID-19 was detected at the provincial level in mainland China during the studied period and that the incidence of COVID-19 was similar among adjacent provinces.

### Spatial distribution

**Fig 2:**
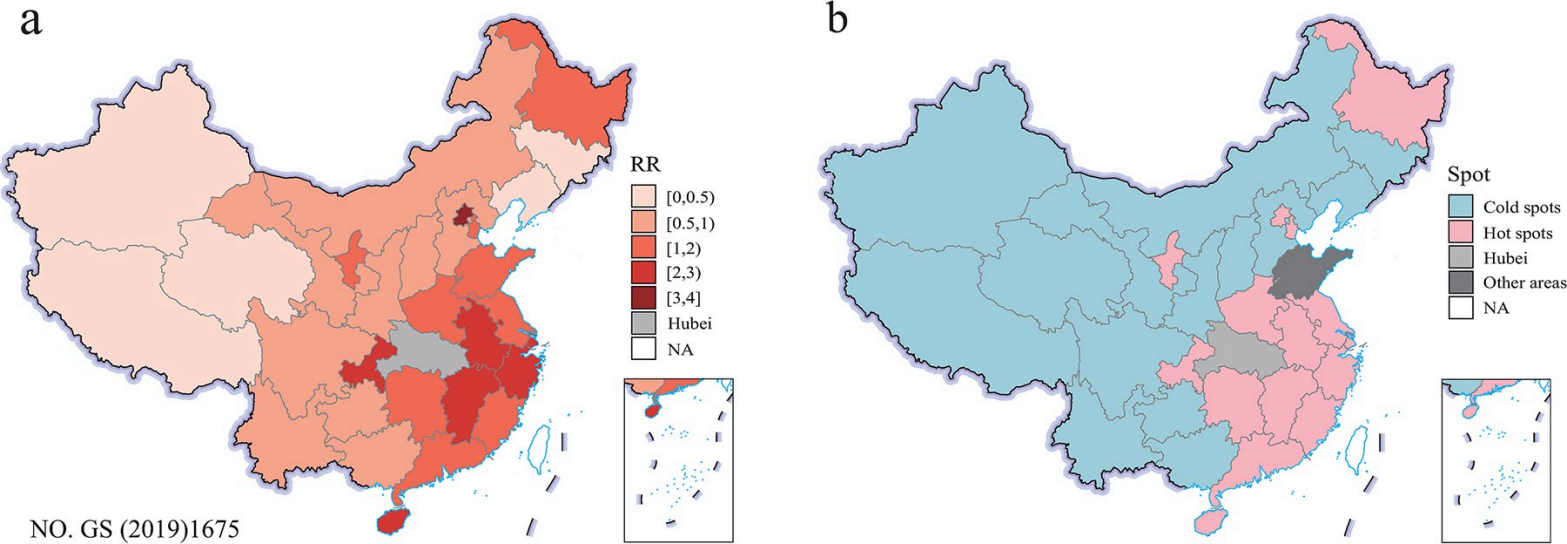
Estimated RR values (a) and hot-cold spots (b) of COVID-19 by Bayesian spatial model without covariables in the 30 provinces in mainland China (outside of Hubei), from 16 January 2020 to 31 March 2020. The original map image was obtained from the China Ministry of Natural Resources(http://bzdt.ch.mnr.gov.cn/index.html).

A Bayesian spatial model without covariates was fitted. The estimated spatial RR (exp(*u*_*i*_+*v*_*i*_)) of COVID-19 by province is shown in Fig 2A, where 14 of 30 provinces had a 95% CI of the RR greater than 1. The high-risk areas were mainly located around Hubei, i.e., Chongqing, Henan, Anhui, Jiangxi, and Hunan, as well as the areas with high economic levels, i.e., Beijing, Zhejiang, Shanghai, Tianjin, and Guangdong. Beijing was the highest risk area, with an RR of 3.57. The provinces with moderate risk indicated by RRs between 2 and 3 were Zhejiang, Shanghai, Jiangxi, Chongqing, Hainan, and Anhui. Notably, the spatial risk was not low in Heilongjiang and Ningxia in northernmost China and northwestern China, respectively. Western and northern China showed a lower risk. Fig 2B maps the distribution of hot-cold spots of COVID-19 in the 30 provinces. Among the 30 provinces, 15 and 14 provinces were classified as hot spots and cold spots, respectively. Shandong was the only province that was neither a hot spot nor a cold spot.

The posterior means of $\sigma_{u}^{2}$ and $\sigma_{v}^{2}$ were 1.277 and 0.002, respectively. The proportion of structured spatial variance in the total variance was 99.75%, indicating that a large part of COVID-19 variability could be explained by the spatial structure. Potential factors associated with the COVID-19 structured spatial variability should be included in the Bayesian spatial model for analysis.

### Relationship of socioeconomic and environmental variables and the incidence of COVID-19

Four variables, i.e., PD, FEEFIT, PCCEOH, and POMPFH, were incorporated into the Bayesian spatial model. The results of the model showed that PCCEOH and POMPFH were significantly and positively correlated with COVID-19. The estimated coefficients for PD and FEEFIT were not significant.

A multivariate Bayesian spatial model with only the significant PCCEOH and POMPFH was fitted. The final coefficients and RR of PCCEOH and POMPFH are listed in Table 2. The results showed that a 10,000 yuan rise in PCCEOH was related to an increase of 88.7% in the COVID-19 risk, and a 1% rise in POMPFH was related to an increase of 9.9% in the COVID-19 risk. The reason for the statistical significance of PCCEOH and POMPFH may be that people from Hubei mainly went to the provinces adjacent to Hubei, and the areas with high economic development experienced more population movements during the Spring Festival travel season.

**Table 2:** The coefficients (*b*) and RR estimated by the multivariate Bayesian spatial model with two socioeconomic variables.

Variable	*b* (95% confidence interval)	RR (95% confidence interval)
Intercept	-1.884 (-2.457, -1.331)	0.158 (0.086, 0.263)
PCCEOH	0.627 (0.384, 0.877)	1.887 (1.469, 2.399)
POMPFH	0.094 (0.052, 0.138)	1.099 (1.053, 1.148)

**Abbreviation**: PCCEOH, per capita consumption expenditure of households; POMPFH, proportion of the migrating population from Hubei from 16 January 2020 to 24 January 2020.

**Fig 3:**
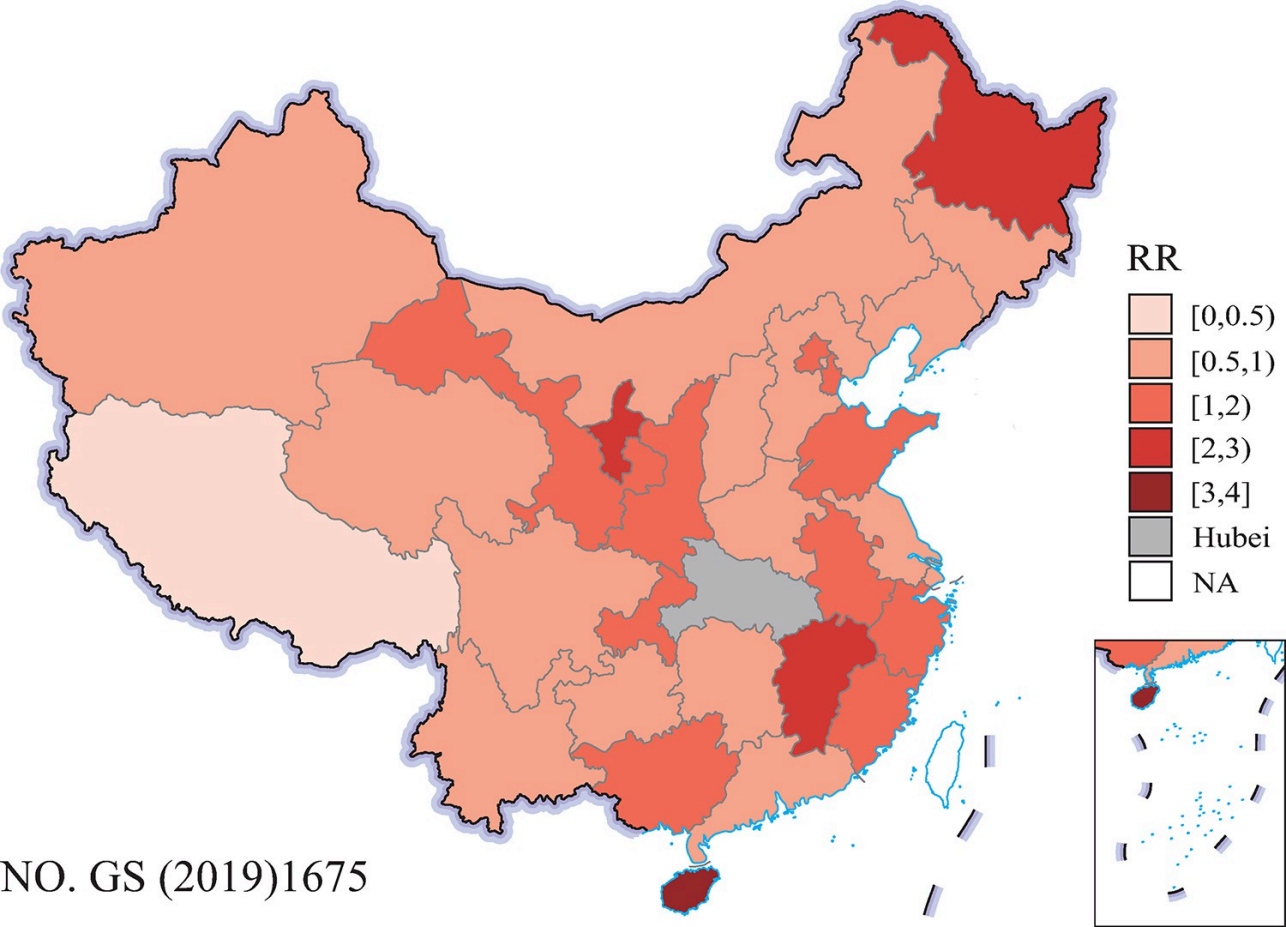
The RR of COVID-19 estimated by the multivariate Bayesian spatial model with two socioeconomic covariables in 30 provinces in mainland China (outside of Hubei), from 16 January 2020 to 31 March 2020. The original map image was obtained from the China Ministry of Natural Resources(http://bzdt.ch.mnr.gov.cn/index.html).

The estimated spatial RR of COVID-19 is shown in Fig 3. Overall, the spatial heterogeneity of RR was reduced, and most RRs were close to 1. Furthermore, the posterior mean of $\sigma_{u}^{2}$ and $\sigma_{v}^{2}$ became 0.002 and 0.227, respectively, and the proportion of structured spatial variance in the total variation was reduced to 21.51%. Thus, the covariables in the model explained 78.24% of the total 99.75% of the structured spatial variance and reduced considerable spatial heterogeneity of RR. However, high RRs were still located in Jiangxi, Ningxia, Hainan, and Heilongjiang. Further research is needed to investigate other variables to explain such excessive risk in these provinces.

## Discussion

From the time it was declared a Public Health Emergency of International Concern (PHEIC) on 30 January 2020, to the time it was declared a global pandemic on 11 March 2020, COVID-19 increasingly threatened the public health security worldwide. This study investigated the spatial distribution of COVID-19 and its relationship with potential influencing factors in China. The results revealed that the areas with a high risk of COVID-19 were in the vicinity of Hubei Province and in the provinces with high economic development, as well as Heilongjiang, Hainan, and Ningxia Province. The proportion of spatial autocorrelation variation in the total variation was reduced from 99.75% to 21.51% by including socioeconomic covariates. Most of the variation in COVID-19 could be explained by socioeconomic factors, which indicated that socioeconomic factors might play important role at the early stage of the pandemic. The per capita consumption expenditure and the proportion of the migrating population from Hubei were positively correlated with the disease risk of COVID-19. These findings can be helpful for the control and prevention of the potential epidemics and emergencies in the future. Several policies such as home quarantine, lockdown, and social distancing were taken to prevent the spread of COVID-19 during the pandemic. These policies varied by regions and their impacts on the COVID-19 pandemic were different. For instance, the traffic blockade in Wuhan city delayed the development of COVID-19 in China [24]. While in others provinces except Hubei, the managements such as home quarantine of migrating population of other regions had played a great role in controlling the local epidemics. However, the effects of these measures were not considered in our study due to the difficulty of quantifying these measures. Further research is needed to explore such impacts of the differences in local policies. Besides, the uncertainty of our study should be mentioned. The COVID-19 cases in our study were collected from the National Health Commission of the People’s Republic of China, which diagnosed and reported timely according to the prevention and control plan of Novel coronavirus pneumonia [25]. The socioeconomic factors were obtained from the China Statistical Yearbook. However, the impacts of the local policies were not considered in this study due to the lack of method to quantify different local policies. Further research is needed to explore the impacts of the local policies.

The high-risk areas of COVID-19 were mainly in eastern and southern China, which was probably associated with population mobility [26, 27]. The COVID-19 outbreak originated in Wuhan, Hubei, China, and coincided with the Spring Festival transport season in 2020. Due to the high infectivity and long incubation period of COVID-19, as well as the lack of prevention and control management before the emergency response was launched, infected individuals and COVID-19 patients spread to other provinces, causing in the COVID-19 epidemic to spread across the provinces that received numerous people from Hubei. From the beginning of the Spring Festival transport season to the 2nd day of the traffic blockade in Wuhan, over half of the outflowing population from Hubei went to adjacent provinces, thus increasing the local risks. The adjacent provinces included Henan, Hunan, Jiangxi, Chongqing, and Anhui. However, high COVID-19 risk was still associated with high per capita consumption expenditure even controlling with the proportion of the migrating population from Hubei. These provinces with high socioeconomic development (Guangdong, Beijing, Shanghai, Zhejiang, and Jiangsu) had higher inflow populations during the Spring Festival transport season than other provinces. The proportion of the population from Hubei in these provinces only reflected the imported population from Hubei. The cases imported through transit might be indirectly reflected in the per capita consumption expenditure.

A study by Wu et al. found independent and self-sustaining community transmission in several major cities in China between 31 December 2019 and 28 January 2020, which might be closely related to the local population density [28]. Notably, the correlation between COVID-19 risk and population density or foreign exchange earnings from international tourism was not statistically significant after controlling for the influence of the proportion of population from Hubei. The reason might be that the implementation of the national emergency response at the end of January 2020 reduced the local COVID-19 exposure by implementing household restrictions and the wearing of masks. Then, the community transmission of COVID-19 was controlled [29, 30], and as were the effects of local population density. This result suggested that the emergency response measures adopted in China sufficiently controlled the community transmission. Besides, the foreign exchange earnings from international tourism partly reflecting the international imported population, were found to be nonsignificant, as there were only a few infected individuals and COVID-19 patients at the early stage. However, with the increasing global threats, more attention should be paid to the risk of imported cases.

High RRs of COVID-19 were found in Jiangxi, Ningxia, Hainan, and Heilongjiang Provinces. The high risks in these four provinces were not explained by the factors considered in this study. One possible reason is the general public’s compliance with the local government’s prevention and control measures during the COVID-19 pandemic. Some COVID-19 patients did not adherence to the prevention measures and even concealed their visits to Wuhan or close contact with people from Wuhan. Such behaviors led to clustered cases and increase the local RR. Several COVID-19 clusters were reported in these four provinces (Jiangxi, Ningxia, Hainan, and Heilongjiang), most of which were family clusters. As of February 6, Heilongjiang had reported 48 clustered outbreaks with 194 cases [31]. Hainan Province had reported 12 clustered outbreaks until February 3 [32]. And there were 43 family clusters cases reported in Ningxia from January 27 to March 16 [33]. As for Jiangxi, a total of 195 clustered outbreaks were reported until February 28 [34].

This study has several limitations. Firstly, the province was the geographic unit used in the spatial analysis. For more useful information and more accurate intervention, a smaller geographic unit scale, such as prefecture level, is needed to analyze in further studies. Secondly, we only explored the relationship between the risk of COVID-19 and socioeconomic factors in this study. Natural and environmental risk factors such as climate and air pollution should be considered in further studies. Thirdly, the impacts of the local policies were not considered in this study due to the lack of method to quantify different local policies. Further research is needed to explore the impacts of the local policies.

## Conclusion

In summary, this study revealed the spatial distribution and investigated influencing factors of COVID-19 in 30 provinces in mainland China (outside of Hubei). The high-risk areas were in the vicinity of Hubei Province and in the provinces with high economic development. The risk of COVID-19 was associated with two socioeconomic factors, the per capita consumption expenditure of households and the proportion of the migrating population from Hubei. Both the model analysis and the actual actions in China showed that blocking population movement and reducing local exposures (by staying at home and wearing masks) were effective in preventing the local transmission of COVID-19.

## Supporting information

S1 Table Spearman correlation coefficients between COVID-19 incidence and each variable of the 30 provinces in mainland China (outsides of Hubei). (PDF)

S1 File (DOCX)
