# Supplementary material for: COVID-19 distributes socially in China: A Bayesian spatial analysis
Source: PLoS One. 2022 Apr 20;17(4):e0267001. doi: 10.1371/journal.pone.0267001 (PMC9020730; doi:10.1371/journal.pone.0267001)
Supplement: S1 Table — (PDF) [file pone.0267001.s001.pdf]

S1 Table. Spearman Correlation Coefficients Between COVID-19 Incidence and Each Variable of the 30 Provinces in Mainland China (outsides of Hubei).

Type	Variable	Spearman
Demographic	Population density	0.65*
	Proportion of urban population at year-end	0.53*
	Percentage of illiterate population to total aged 15 and over	-0.28
Economic	Gross domestic product	0.46*
	Foreign exchange earnings from international tourism	0.43*
	Per capita consumption expenditure of households	0.54*
Health	Number of health care institutions	-0.07
	Number of beds in health care institutions	0.14
	Medical technical personnel in health care institutions per 1000 persons	0.09
	Beds of medical institutions per 1000 population	-0.26
Transportation	Passenger traffic	0.31
	Passenger kilometers	0.20
	Proportion of the migrating population from Hubei from 16 January 2020 to 24 January 2020	0.57*

*P<0.05.
