# Supplementary material for: COVID-19 distributes socially in China: A Bayesian spatial analysis
Source: PLoS One. 2022 Apr 20;17(4):e0267001. doi: 10.1371/journal.pone.0267001 (PMC9020730; doi:10.1371/journal.pone.0267001)
Supplement: S1 File — (DOCX) [file pone.0267001.s002.docx]

The maps images in Figures 1-3 were obtained from the China Ministry of Natural Resources(<http://bzdt.ch.mnr.gov.cn/index.html>). According to official note, the public can use these maps marked the map approval number directly. We have marked the map approval number in Figures 1-3(NO. GS (2019)1675).
